# Proximity-aware interactive displays for rehabilitation centres

**DOI:** 10.1080/07853890.2021.1897439

**Published:** 2021-09-28

**Authors:** Afonso Faria, Stéphane Duarte, Daniel Simões Lopes, Hugo Nicolau

**Affiliations:** INESC-ID, Instituto Superior Técnico, Universidade de Lisboa, Lisbon, Portugal

## Abstract

**Introduction:**

In clinical practice, physiotherapists often support 3–5 patients, simultaneously. They are frequently roaming throughout the room, switching between patients, taking notes, planning, demonstrating, coordinating, and monitoring multiple exercises. In such demanding environments, it is common for important events to go unnoticed. In light of this, context-aware computing – the ability to recognise people/activities and present timely information – can support rehabilitation practices for both clinicians and patients. Previous research proposed augmenting everyday objects to aid medical professionals [1] or using large interactive whiteboards that show situational information in operating rooms [2]. Nevertheless, the potential of context-aware computing remains largely unexplored in rehabilitation spaces as technologies fail to support the dynamic nature of clinical settings. In this work, we propose ARCADE, a proximity-aware system that leverages motion tracking and interactive displays to support patients’ rehabilitation and provide meaningful and timely information to physiotherapists. The design of the system is grounded on the theory of proxemics [3]; particularly, we leveraged the concept of interpersonal distance (i.e. relative distance between two people) to adjust the information being displayed to both patient and professional. ARCADE is sensitive to 3 interpersonal distances: *intimate* (<0.5 m), *personal* (0.5–1.5m), and *social* (>1.5 m). When therapists are attending other patients (*social*), the information displayed can be adequately be seen from a distance, showing progress and whether the patient is performing the exercises correctly. When therapists move towards the patient (*personal*), the display changes smoothly to show critical performance information. Furthermore, therapists can use their hand as a virtual stethoscope to display detailed measures about a specific body segment/joint (*intimate*). The information being displayed at each interpersonal distance emerged from field studies with physiotherapists from local rehabilitation institutions. The user study aimed to answer two main research questions: (1) is ARCADE effective in illustrating the patients’ performance in unsupervised exercises? and (2) are proxemic interactions useful in rehabilitation environments?

**Materials and methods:**

We conducted an evaluation with 9 physiotherapists, using both quantitative and qualitative methods to understand the system’s potential to be used in clinical settings. The study had two stages. First, we demonstrated ARCADE to participants and used a think-aloud protocol to elicit feedback about its usefulness in clinical settings. We then conducted a thematic analysis to all qualitative data. In the second stage, we simulated a situation where patients perform an exercise while unsupervised. We compared therapists’ performance in scoring the quality of movement using ARCADE’s visualisations against real-time video performance of patients. ARCADE’s visualisations used a human body representation with 20 joints and included measures such as task completion rate, joint angles, body segment paths, number of compensatory movements, and common compensations. The visualisations illustrated patients’ unsupervised performance based on the video recordings.

**Results:**

Therapists responded positively to the visual measures displayed by ARCADE, and how information changed based on proximity. Interestingly, they were able to combine multiple measures to assess patients’ performance and uncover hidden information that is not visible by the human eye (e.g. joint angles and compensation movements). Results from stage 2 showed that post-assessment of patients’ performance using ARCADE was similar to physiotherapists’ real-time observations in terms of movement speed, amplitude, precision and overall movement quality.

**Discussion and conclusions:**

We present ARCADE, a novel proximity-aware system that displays meaningful and timely information to patients and physiotherapists. ARCADE demonstrated potential to be used as a rehabilitation tool and enable professionals to assess the performance of multiple patients, simultaneously, without individual performance loss.
